# Novel Microchip-Based Tools Facilitating Live Cell Imaging and Assessment of Functional Heterogeneity within NK Cell Populations

**DOI:** 10.3389/fimmu.2012.00300

**Published:** 2012-10-05

**Authors:** Elin Forslund, Karolin Guldevall, Per E. Olofsson, Thomas Frisk, Athanasia E. Christakou, Martin Wiklund, Björn Önfelt

**Affiliations:** ^1^Department of Microbiology, Tumor and Cell Biology, Karolinska InstituteStockholm, Sweden; ^2^Department of Applied Physics, KTH – Royal Institute of TechnologyStockholm, Sweden

**Keywords:** NK cell, live cell imaging, cytotoxicity, microchip, ultrasound, cell migration, single cell

## Abstract

Each individual has a heterogeneous pool of NK cells consisting of cells that may be specialized towards specific functional responses such as secretion of cytokines or killing of tumor cells. Many conventional methods are not fit to characterize heterogeneous populations as they measure the average response of all cells. Thus, there is a need for experimental platforms that provide single cell resolution. In addition, there are transient and stochastic variations in functional responses at the single cell level, calling for methods that allow studies of many events over extended periods of time. This paper presents a versatile microchip platform enabling long-term microscopic studies of individual NK cells interacting with target cells. Each microchip contains an array of microwells, optimized for medium or high-resolution time-lapse imaging of single or multiple NK and target cells, or for screening of thousands of isolated NK-target cell interactions. Individual NK cells confined with target cells in small microwells is a suitable setup for high-content screening and rapid assessment of heterogeneity within populations, while microwells of larger dimensions are appropriate for studies of NK cell migration and sequential interactions with multiple target cells. By combining the chip technology with ultrasonic manipulation, NK and target cells can be forced to interact and positioned with high spatial accuracy within individual microwells. This setup effectively and synchronously creates NK-target conjugates at hundreds of parallel positions in the microchip. Thus, this facilitates assessment of temporal aspects of NK-target cell interactions, e.g., conjugation, immune synapse formation, and cytotoxic events. The microchip platform presented here can be used to effectively address questions related to fundamental functions of NK cells that can lead to better understanding of how the behavior of individual cells add up to give a functional response at the population level.

## Introduction

Patrolling peripheral blood and residing in secondary lymphoid organs, NK cells are part of the first line of defense against infections and tumors. Without need for prior immunization, NK cells are capable of direct cytotoxicity and also influence other aspects of the immune system through secretion of cytokines (Trinchieri, 1989). Human NK cell populations have been divided into two major phenotypic subsets; the CD56^bright^ and CD56^dim^ subsets which display differences in their functional responses (Nagler et al., 1989; Jacobs et al., 2001). Cells belonging to the CD56^bright^ subset are enriched in secondary lymphoid organs where they upon stimulation proliferate rapidly and secrete cytokines more readily than CD56^dim^ cells (Cooper et al., 2001a,b). More recently it was confirmed that CD56^bright^ cells are precursors of CD56^dim^ cells, which have more cytolytic activity (Ferlazzo et al., 2004; Romagnani et al., 2007). A unique characteristic of NK cells is the ability to detect and selectively kill cells that have compromised expression of MHC class I molecules (Kärre et al., 1986). This “missing self” recognition of MHC is facilitated in humans through their killer cell immunoglobulin-like receptors (KIRs), which are functionally analogous to murine Ly49 receptors, and lectin-like CD94/NKG2 receptors present in both species (Ljunggren and Karre, 1990; Parham, 2005). Significant heterogeneity has been observed within the CD56^dim^ population in terms of KIR expression and missing self recognition, indicating the presence of functional subsets yet to be identified (Yawata et al., 2008). Moreover, repertoires of MHC class I receptors have been shown to correlate with the strength of the cytotoxic response (Brodin et al., 2009). Expression of NK cell receptors not only varies within populations but also significantly between individuals and over time (Valiante et al., 1997; Bjorkstrom et al., 2010), highlighting the complexity of NK cell responses. Clearly, NK cell populations are heterogeneous, comprising individual cells with differences in their cytotoxic potential.

## Conventional Methods for Assessment of NK Cell Heterogeneity

Many conventional population-based methods estimate the average distribution of responses and do not provide sufficient information of the potency and efficacy of individual cells. One exception to this is flow cytometry, a widely used technique that allows for multiparametric analysis with single cell resolution. Flow cytometric analysis permits accurate detection of biomarkers, and it can also be used to analyze functional responses by for example staining for intracellular cytokines or the degranulation marker CD107a. Although the analysis is at the single cell level, the responses are still often measured subsequent of activation in bulk culture. A further limitation of flow cytometry is the fixed time of assessment and consequent lack of information regarding dynamics and transient fluctuations in cellular responses. Dynamic processes such as effector-target cell interactions and cell migration can be assessed by live cell imaging. However, conventional microscopic approaches are often inefficient (in particular for studies of suspension cells) as the cells often move out of the field of view and therefore cannot be followed for extended periods of time. Moreover, to resolve heterogeneity of cell populations, the number of observations needs to be considerably higher than what is normally achieved with a conventional approach to live cell imaging.

## Novel Microchip-Based Methods with Single Cell Resolution

In recent years several lab-on-a-chip devices have been used for single cell studies (Lindstrom and Andersson-Svahn, 2012). This includes studies of fibroblasts, stem cells, and B cells (Chin et al., 2004; Rettig and Folch, 2005; Yamamura et al., 2005; Tokimitsu et al., 2007). Still, relatively few studies have focused on microchip-based culture and functional assessment of NK cells (Guldevall et al., 2010; Khorshidi et al., 2011; Yamanaka et al., 2012). Since NK cells as well as T cells have high migratory capacity, the confinement of these cells in microwells becomes more challenging as compared to many other cell types. Successful approaches for studying functional NK or T cell responses include trapping cells between a PDMS microwell and a coverglass, with subsequent characterization of cytokine secretion, cytotoxicity, or gene expression of single cells (Love et al., 2006; Gong et al., 2010; Han et al., 2010, 2012; Yamanaka et al., 2012). Microwell devices have also been combined with micropatterning techniques, allowing T cells to attach to cell-adhesive ligands in microwells (Revzin et al., 2005).

**Figure 1:**
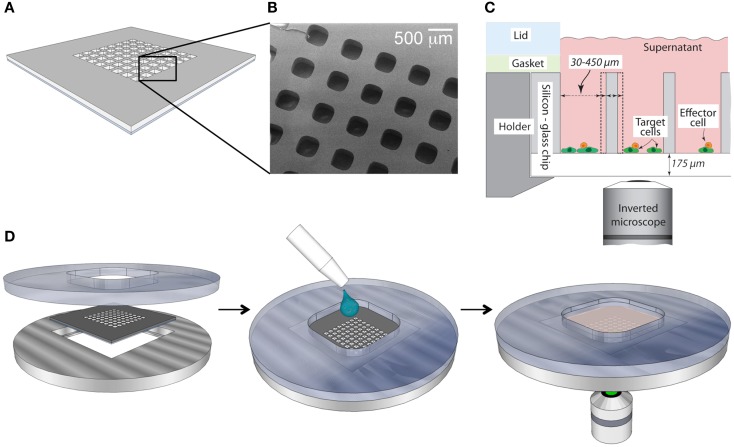
**Schematic overview of the microchip platform**. **(A)** The silicon microchip is made of a 22 mm× 22 mm silicon wafer with microwells of variable dimensions etched in the center. After etching the mesh is bonded to a thin glass slide, providing transparent well bottoms suitable for imaging in inverted microscopes. **(B)** Scanning electron microscope micrograph of a small section of microchip with square wells (sides 300 μm) depicted from above. **(C)** Cross-sectional schematic view of the loaded chip when positioned inside the stainless steel/plastic holder. The well dimensions can be varied to fit individual experimental set-ups. **(D)** Device assembly (left), sample loading (middle), and image acquisition (right).

The microchip platform presented in this paper (Figure 1) enables multidimensional, long-term microscopic studies of individual NK cells (Frisk et al., 2011). Because of the large depth of each well (300 μm), motile cells are safely trapped and unable to move between the microwells. Confinement of cells enables us to follow single cells over extended periods of time. Furthermore, our microwell imaging-platform is versatile, where the size of the wells can be chosen according to the study. The microchips, which are made out of silicon and glass, are biocompatible and provide an *in vitro* environment comparable to that of standardized cell cultures (Guldevall et al., 2010). Seeding of the chip is performed by adding cell suspension on top of the chip and allowing cells to sediment into the wells (Figure 1D). The total number of cells needed for one experiment is at the most 100,000, making the system appropriate for studies where the sample size is limited. The resulting distribution of cells over the chip depends on the initial cell density, and added volume, which are chosen to achieve an appropriate cell distribution for any given experimental setup. The microchip is easily mounted in a holder that fits conveniently onto regular microscope stages. Since the bottom of the chip has the same thickness as a regular coverglass, high-resolution imaging of the wells is straightforward.

### Small wells for screening and time-lapse imaging

**Figure 2:**
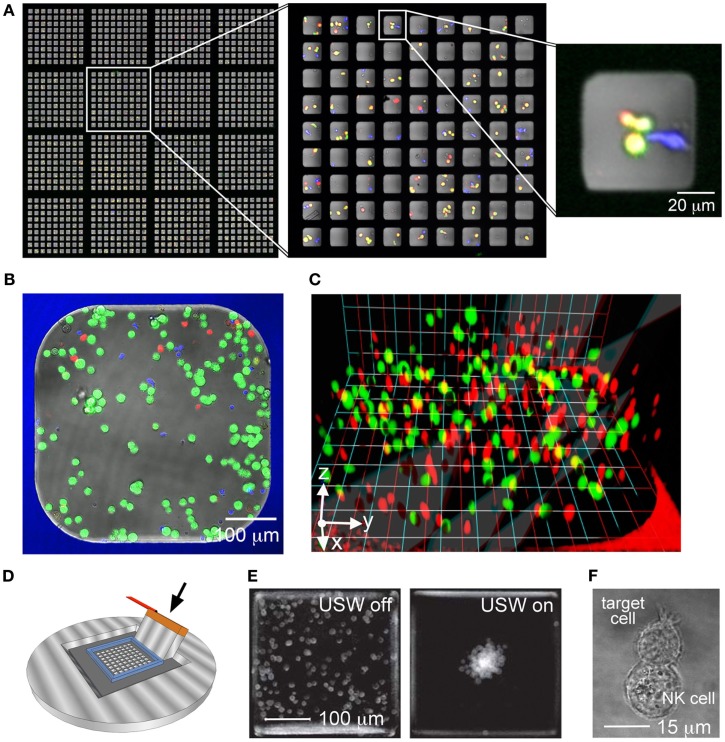
**The microchip platform provides a versatile base for several experimental set-ups**. **(A)** Partial overview of a microchip containing 32 400 square wells with 50 μm sides seeded with effector and target cells. The chip contains 20 × 20 sections with 9 × 9 wells each (the left image shows 4 × 4 such sections). The sections with 9 × 9 wells (middle image) are fabricated to fit in the field of view of a 10× microscope objective, providing enough resolution to observe cells inside individual wells (right). **(B)** Slightly larger well (450 μm sides) used for 2D migration and cytotoxicity studies loaded with a small population of murine NK cells (blue) and target cells labeled with the viability dye calcein (green) and cell tracer dye DDAO (red). Dead target cells show up as bright red. **(C)** Human primary NK cells (red) and tumor target cells (green) loaded in a collagen gel inside a microwell forming an *in vivo*-mimicking 3D matrix for migration and cytotoxicity studies. **(D)** Schematic image of the ultrasonic wave (USW) device. A transducer (arrow) glued to the silicon microchip produces ultrasonic standing waves in the wells. **(E)** Distribution of cells in several superimposed microwells with the ultrasound turned off (left) or turned on (right). Image reproduced from Vanherberghen et al. (2010). **(F)** Example of a conjugate between human NK cell (YTS) and B cell (721.221) formed inside a microwell in the USW device.

Microwells of 50 × 50 × 300 μm (side × side × depth) are designed to fit only a few cells inside each well (Figure 2A). Screening of a large array of these microwells can be used to assess functional heterogeneities, such as cytotoxicity, within NK cell populations (Guldevall et al., 2010). The minimal distance separating the target and effector cells in the wells increases the probability of cell–cell interactions, hence improving experimental efficiency. Moreover, the sheer number of wells, providing up to 100,000 observations in one experiment, strengthens the statistical impact of the study and allows for accurate detection of rare events. When screening for killing events, the chip is first seeded with target and effector cells and imaged. The chip is then incubated for some time and finally imaged again. An automatic image analysis software (Frisk et al., 2011) permits finding and counting all NK cells, as well as living and dead target cells before and after the incubation. Here, wells containing target cells alone serve as an intrinsic control for spontaneous target cell death. In this way, killing events are detected and individual NK cells can be ascribed different cytotoxic potency. A similar single cell cytolysis assay has recently been developed to study cytotoxic T cell clones from HIV patients as well as NK cell heterogeneity (Varadarajan et al., 2011; Yamanaka et al., 2012). A great benefit with that method is that it also allows detection of cytokine secretion from individual cells (microengraving) but a drawback is that the cells cannot stay trapped in the device longer than a few hours before starting to die (Love et al., 2006; Guldevall et al., 2010). The considerable well depth of 300 μm and large open volume above the microchip presented here ensures entrapment of the cells and supply of fresh cell media permitting long-term (>4 days) experiments (Guldevall et al., 2010; Frisk et al., 2011). Thus, this allows for read-outs like, e.g., clonal expansion of activated cells. These small wells can also be used to follow cell–cell interaction events by time-lapse microscopy. However, the number of wells that can be followed in parallel is limited by the desired time resolution and image quality.

### Larger wells for studies of killing and migration in 2D and 3D

Immune surveillance performed by NK cells involves migration within and across different tissues including blood, spleen, and lungs. The migratory capacity of NK cells can be characterized using our larger microwells of 300–900 μm that are well suited for studies of small populations of cells (but with maintained single cell resolution) over extended time periods (Khorshidi et al., 2011). For convenient tracking of cell migration, about 50–100 NK cells and 100–200 target cells are loaded into each microwell (Figure 2B). More than 10 wells can be imaged in parallel and still provide adequate resolution for image-analysis given that a fluorescence microscope with an automated stage is used. Simultaneous recording of events in several wells not only increases the number of cells that are assessed but also allows for comparison of different NK cell populations. Thus, this setup allows systematic studies of migratory and killing behaviors of different NK cell populations addressing questions related to, e.g., tumor surveillance or NK cell education and tolerance (Forslund et al., 2012; Vanherberghen et al., 2012). Cell migration is much dependent on the environment and the extracellular matrix (ECM) provides mechanical support for cell anchorage that in turn aids cell motility. To create a more *in vivo* like environment, the microwells can be coated with ECM proteins such as fibronectin. It is also possible to load the whole microwell volume with a collagen gel that supports 3D migration (Figure 2C). In an ongoing study of human NK cell migration in a collagen matrix we have found that NK cells maintain their cytotoxicity in the matrix and that they migrate faster in 3D compared to 2D (Olofsson et al., 2012). This difference in migration may suggest a more *in vivo* like behavior as murine NK cells imaged in lymph nodes have been observed to migrate faster compared to both human and murine NK cells imaged in 2D cultures *in vitro*. Thus, this 3D system could be a valuable tool for studies of migration and cytotoxicity of individual NK cells under controlled conditions, especially for human systems where *in vivo* imaging is impossible.

### Ultrasound induced cell–cell contact in multiwell chips

In addition to migratory behavior, it is of great interest to study the dynamic interactions taking place subsequent to the initial cell–cell contact. However, in sparse *in vitro* cultures the time required for an NK cell to spontaneously find and make contact with a target cell can extend over hours, even if the cells are confined in small wells (Guldevall et al., 2010). We have recently developed an ultrasound-based method for inducing and synchronizing the interaction between an NK cell and the target cells in multiple parallel microwells (Vanherberghen et al., 2010; Christakou et al., 2012; Wiklund and Onfelt, 2012). This ultrasonic method efficiently induces cell–cell contacts in all wells shortly after the ultrasound has been turned on (within seconds), greatly facilitating time-lapse studies of NK-target cell interactions. The principle is based on acoustic radiation forces from a resonant ultrasonic field (Bruus, 2012). Upon ultrasonic actuation, cells move into the pressure nodes of the standing wave where they are aggregated and trapped. In contrast to other available physical methods for cell manipulation (e.g., dielectrophoresis and optical tweezers; Neuman et al., 1999; Muller et al., 2003) the method has been shown to be particularly suitable for gently merging and aggregating cells with minimal negative impact on the cellular state and viability (Wiklund, 2012). The ultrasound can be coupled into the wells by simply vibrating the chip with a piezoelectric transducer (Figure 2D). The frequency is chosen in order to set up a standing wave in each microwell. A single trapping position for the cell cluster in the center of the well is achieved by matching the well dimension with half the wavelength of the acoustic wave. For example, a 300 μm well corresponds to an actuation frequency of 2.5 MHz. The uniformity and robustness of the trapping and positioning method is further enhanced by driving the transducer with frequency sweeps around this center frequency, e.g., by linear sweeps from 2.45 to 2.55 MHz at a rate of typically 1 kHz (Vanherberghen et al., 2010). A typical cell clustering result in a 100-well chip actuated by ultrasound is seen in Figure 2E. The rapid formation of NK-target cell clusters by ultrasound in microwells is useful for time-lapse confocal microscopy since the vast majority of NK-target cell interactions in the experiment are synchronized, i.e., having the same starting time. Furthermore, the accurate and robust positioning of the cell clusters in the ultrasound field facilitates high-resolution imaging, something that has been used for characterization of the interactions between NK cells and target cells (Figure 2F). This ultrasound method is currently used for quantifying the heterogeneity of the cytotoxic response of NK cells against tumor target cells (Christakou et al., 2012).

### Labeling of cells and detection of killing

**Figure 3:**
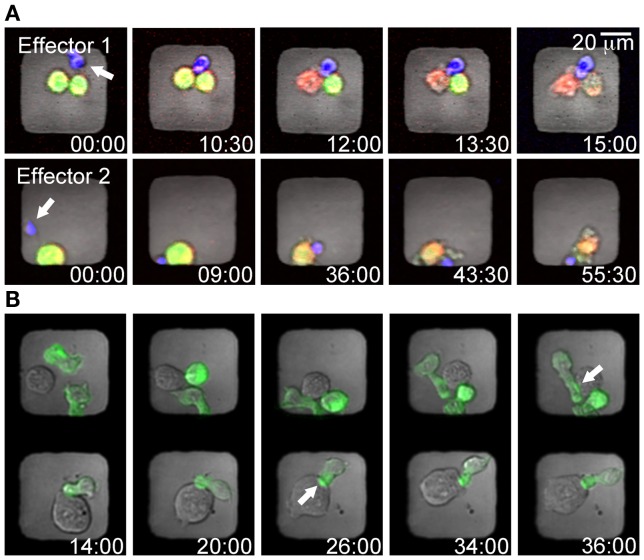
**Examples of cell labeling strategies to detect NK mediated cytotoxicity or cell–cell interactions in microwells**. **(A)** Time-lapse series showing two wells containing individual NK cells (blue) killing tumor target cells (yellow). Target cells were stained with the cytosolic dye calcein (green) and the red cell tracer dye DDAO (red); hence the killing events can be detected by lysed target cells changing color from yellow to red. **(B)** To detect the dynamics of immune synapse formation B cells expressing GFP-labeled MHC I molecules (721.221/Cw6-GFP, green) were co-incubated with NK cells (YTS/KIR1, unstained). The timing of immune synapse formation in cell conjugates trapped in adjacent wells was assessed by monitoring clustering of the MHC I-protein (green) at the intercellular surface (white arrows).

Target and effector cells should be labeled separately with different fluorescent dyes before being seeded into the microchip. Selective labeling not only helps distinguishing the two cell types but also enables detection of cell death and subsequent identification of killing events. There are many ways of labeling cells with dyes that indicate viability. One example is calcein which during labeling diffuse across the plasma membrane and is retained in the cytosol as a result of intracellular enzyme cleavage. When the plasma membrane is compromised, the calcein dye leaks out of the cell (Figure 3A). Leakage of the dye can be followed and plotted against time, where the gradient of the declining curve indicates the rate of cell death (Guldevall et al., 2010). To identify target cells that were already dead when seeding the chip and to further improve detection of killing, target cells can be labeled with an additional dye that should be independent of membrane integrity. As a result, a killing event can be identified when lysed target cells change color, e.g., from green to red (Figure 3A). In addition to analysis of calcein leakage, target cell death can also be recognized by changes in membrane morphology, usually characterized by membrane blebbing visible in the bright-field channel. Due to the thin glass in the bottom of the microwells the microchip platform is also suitable for high-resolution imaging of subcellular events such as the dynamics of protein accumulation in the inhibitory immune synapse (Figure 3B).

### Analysis of migration behavior

**Figure 4:**
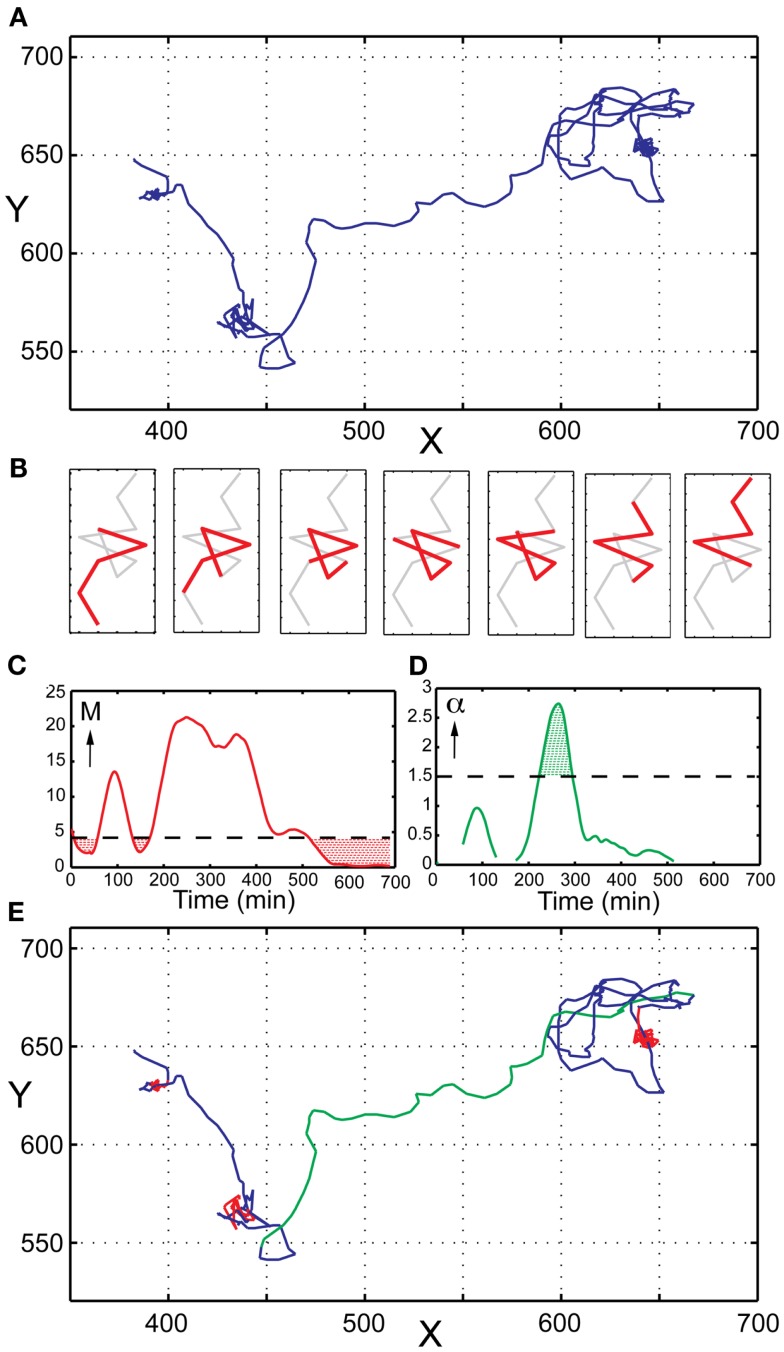
**Analysis of transient migration behavior**. **(A)** Trajectory of a single human primary NK cell imaged in a microwell. **(B)** Schematic representation of the sliding window approach to migration analysis. Only a small part of the trajectory is analyzed at a time (here represented by five consecutive point shown in red). Step-by-step a new segment is analyzed until the whole trajectory has been covered. **(C)** TMAPs occur when *M* is below the threshold value (red dotted areas). **(D)** Directed migration occurs when α is higher than the chosen threshold value (here α = 1.5) for 10 successive time points. **(E)** Same NK cell trajectory as in **(A)** with indicated modes of migration; random movement (blue), TMAPs (red), and directed migration (green).

Heterogeneity in terms of migration behavior can be quantified by imaging NK cells confined in microwells followed by single cell tracking, thus creating distinct trajectories for all NK cells (Figure 4A). From these trajectories it is possible to calculate and compare parameters such as speed, displacement, and direction for each cell. Recent data have shown that NK cells, similar to T cells display transient variations in their migratory behavior. NK cells commonly display a stop and go behavior, alternating between periods of fast and slow migration as well as complete migration arrest. The factors regulating the transitions between such periods are poorly understood even if it has been shown that interactions with target cells make NK cells slow down (Khorshidi et al., 2011), especially for NK cells producing interferon-γ (Yamanaka et al., 2012). Furthermore, it has been shown that NK cell stopping is coupled to ligation of activating receptors (Culley et al., 2009). In order to facilitate analysis of NK cell time-lapse imaging data we recently developed a method detecting transient variations in migration behavior (Khorshidi et al., 2011), which is briefly described below.

A central parameter in single particle tracking is mean-square displacement (MSD), and is widely used as a means of estimating the diffusion coefficient. For single cell tracking the same parameter can be used to characterize migratory behaviors but then the diffusion coefficient is instead called the migration coefficient. The MSD is a measure related to the average distance migrated and can be written analytically as 〈MSD(*t*)〉 = 4*Mt*^α^ for 2D migration, and 〈MSD(*t*)〉 = 6*Mt*^α^ for 3D migration. Here *M* is the migration coefficient and the value of α can be used to determine if the migration is directed or random. To reveal transient behavior in NK cell trajectories, we use a sliding window approach, where only a small part of the trajectory is evaluated at a time (Figure 4B). Step-by-step the values of *M* and α are determined for each segment until the whole trajectory is analyzed. The estimated diffusion coefficient of an NK-cell-sized particle undergoing Brownian motion serves as a threshold for periods characterized by low motility. At any time where the migration coefficient is smaller than this threshold, the cell is defined to be in a transient migratory arrest period (TMAP; Figure 4C). Cell movement can be further classified as ‘random movement’ if the cell is outside a TMAP and α ≤1.5. If α >1.5 for at least 10 successive time points, cells are classified to migrate in a directed fashion (Figure 4D). Directed migration is characterized by persistent and highly correlated motion, usually in a single direction. A trajectory with all above-mentioned modes of migration indicated can be viewed in Figure 4E, emphasizing the transient behavior displayed by NK cells.

This analytic approach resulted in several interesting findings about NK cell behavior. For example, *in situ* data revealed that NK cells spend more time in directed migration during inflammatory conditions. Moreover, formation of NK-target cell conjugates often coincided with detection of TMAPs. Interestingly, the area covered by TMAPs was often similar to the size measured for target cells (Khorshidi et al., 2011). Hence, this analytical method together with our microwell based assay can serve as useful tool in determining the effects of different agents or conditions on the migration of individual NK cells. The analysis could also be useful for *in vivo* imaging data where it is difficult to detect interactions between fluorescently labeled cells and unlabeled cells.

## Conclusion

A population of NK cells is composed of individual cells with different strength and efficacy in their effector responses. Dissection of this functional heterogeneity requires an analytical platform that allows for efficient studies at the single cell level over time. With the microchip platform presented here, cytotoxicity, migration, and proliferation can be assessed for thousands of parallel NK-target cell conjugates in a single experiment. This and similar analytical platforms are currently establishing as valuable tools for modern cell biology and immunology.

## Conflict of Interest Statement

Björn Önfelt, Thomas Frisk, Martin Wiklund are inventors of patents related to the technology presented.

## References

[B1] Bjorkstrom N. K. Riese P. Heuts F. Andersson S. Fauriat C. Ivarsson M. A. (2010). Expression patterns of NKG2A, KIR, and CD57 define a process of CD56dim NK-cell differentiation uncoupled from NK-cell education. Blood 116, 3853–3864 10.1182/blood-2010-04-281675 20696944

[B2] Brodin P. Lakshmikanth T. Johansson S. Karre K. Hoglund P. (2009). The strength of inhibitory input during education quantitatively tunes the functional responsiveness of individual natural killer cells. Blood 113, 2434–2441 10.1182/blood-2008-05-156836 18974374

[B3] Bruus H. (2012). Acoustofluidics 7: the acoustic radiation force on small particles. Lab Chip 12, 1014–1021 10.1039/c1lc20770a 22349937

[B4] Chin V. I. Taupin P. Sanga S. Scheel J. Gage F. H. Bhatia S. N. (2004). Microfabricated platform for studying stem cell fates. Biotechnol. Bioeng. 88, 399–415 10.1002/bit.20254 15486946

[B5] Christakou A. Ohlin M. Khorshidi M. A. Kadri N. Vanherbergen B. Frisk T. (2012). “Ultrasound mediated cell aggregation in multiwell microchips facilitates studies of interactions between natural killer cells and target cells,” in Poster Presented at the 13th Meeting of the Society for Natural Immunity, Asilomar, CA

[B6] Cooper M. A. Fehniger T. A. Caligiuri M. A. (2001a). The biology of human natural killer-cell subsets. Trends Immunol. 22, 633–640 10.1016/S1471-4906(01)02060-9 11698225

[B7] Cooper M. A. Fehniger T. A. Turner S. C. Chen K. S. Ghaheri B. A. Ghayur T. (2001b). Human natural killer cells: a unique innate immunoregulatory role for the CD56(bright) subset. Blood 97, 3146–3151 10.1182/blood.V97.10.3146 11342442

[B8] Culley F. J. Johnson M. Evans J. H. Kumar S. Crilly R. Casasbuenas J. (2009). Natural killer cell signal integration balances synapse symmetry and migration. PLoS Biol. 7, e1000159 10.1371/journal.pbio.1000159 19636352 PMC2707003

[B9] Ferlazzo G. Thomas D. Lin S. L. Goodman K. Morandi B. Muller W. A. (2004). The abundant NK cells in human secondary lymphoid tissues require activation to express killer cell Ig-like receptors and become cytolytic. J. Immunol. 172, 1455–1462 14734722 10.4049/jimmunol.172.3.1455

[B10] Forslund E. Kadri N. Frisk T. Kärre K. Höglund P. Önfelt B. (2012). “Characterization of murine NK cell migration and killing of MHC class I deficient target cells at the single cell level,” in Poster presented at The 13th meeting of the Society for Natural Immunity, Asilomar, CA

[B11] Frisk T. W. Khorshidi M. A. Guldevall K. Vanherberghen B. Onfelt B. (2011). A silicon-glass microwell platform for high-resolution imaging and high-content screening with single cell resolution. Biomed. Microdevices 13, 683–693 10.1007/s10544-011-9538-2 21465090

[B12] Gong Y. Ogunniyi A. O. Love J. C. (2010). Massively parallel detection of gene expression in single cells using subnanolitre wells. Lab Chip 10, 2334–2337 10.1039/c004847j 20686711 PMC4040084

[B13] Guldevall K. Vanherberghen B. Frisk T. Hurtig J. Christakou A. E. Manneberg O. (2010). Imaging immune surveillance of individual natural killer cells confined in microwell arrays. PLoS ONE 5, e15453 10.1371/journal.pone.0015453 21103395 PMC2980494

[B14] Han Q. Bagheri N. Bradshaw E. M. Hafler D. A. Lauffenburger D. A. Love J. C. (2012). Polyfunctional responses by human T cells result from sequential release of cytokines. Proc. Natl. Acad. Sci. U.S.A. 109, 1607–1612 10.1073/pnas.1206828109 22160692 PMC3277116

[B15] Han Q. Bradshaw E. M. Nilsson B. Hafler D. A. Love J. C. (2010). Multidimensional analysis of the frequencies and rates of cytokine secretion from single cells by quantitative microengraving. Lab Chip 10, 1391–1400 10.1039/c005296e 20376398 PMC3128808

[B16] Jacobs R. Hintzen G. Kemper A. Beul K. Kempf S. Behrens G. (2001). CD56bright cells differ in their KIR repertoire and cytotoxic features from CD56dim NK cells. Eur. J. Immunol. 31, 3121–3127 10.1002/1521-4141(2001010)31:10<3121::AID-IMMU3121>3.0.CO;2-4 11592089

[B17] Kärre K. Ljunggren H. G. Piontek G. Kiessling R. (1986). Selective rejection of H-2-deficient lymphoma variants suggests alternative immune defence strategy. Nature 319, 675–678 10.1038/319675a0 3951539

[B18] Khorshidi M. A. Vanherberghen B. Kowalewski J. M. Garrod K. R. Lindstrom S. Andersson-Svahn H. (2011). Analysis of transient migration behavior of natural killer cells imaged in situ and in vitro. Integr. Biol. (Camb.) 3, 770–778 10.1039/c1ib00007a 21687858

[B19] Lindstrom S. Andersson-Svahn H. (2012). Single-Cell Analysis Methods and Protocols. Methods in Molecular Biology 853, ed. Walker J. M. (New York: Humana Press). 10.1007/978-1-61779-567-1 22420047

[B20] Ljunggren H. G. Karre K. (1990). In search of the ‘missing self’: MHC molecules and NK cell recognition. Immunol. Today 11, 237–244 10.1016/0167-5699(90)90097-S 2201309

[B21] Love J. C. Ronan J. L. Grotenbreg G. M. Van Der Veen A. G. Ploegh H. L. (2006). A microengraving method for rapid selection of single cells producing antigen-specific antibodies. Nat. Biotechnol. 24, 703–707 10.1038/nbt1210 16699501

[B22] Muller T. Pfennig A. Klein P. Gradl G. Jager M. Schnelle T. (2003). The potential of dielectrophoresis for single-cell experiments. IEEE Eng. Med. Biol. Mag. 22, 51–61 10.1109/MEMB.2003.1266047 15007991

[B23] Nagler A. Lanier L. L. Cwirla S. Phillips J. H. (1989). Comparative studies of human FcRIII-positive and negative natural killer cells. J. Immunol. 143, 3183–3191 2530273

[B24] Neuman K. C. Chadd E. H. Liou G. F. Bergman K. Block S. M. (1999). Characterization of photodamage to Escherichia coli in optical traps. Biophys. J. 77, 2856–2863 10.1016/S0006-3495(99)77117-1 10545383 PMC1300557

[B25] Olofsson J. Frisk T. Önfelt B. (2012). “Time-lapse imaging of NK cell migration in 3D collagen matrices formed in microwells,” in Poster Presented at the 13th Meeting of the Society for Natural Immunity, Asilomar, CA

[B26] Parham P. (2005). MHC class I molecules and KIRs in human history, health and survival. Nat. Rev. Immunol. 5, 201–214 10.1038/nri1570 15719024

[B27] Rettig J. R. Folch A. (2005). Large-scale single-cell trapping and imaging using microwell arrays. Anal. Chem. 77, 5628–5634 10.1021/ac0505977 16131075

[B28] Revzin A. Sekine K. Sin A. Tompkins R. G. Toner M. (2005). Development of a microfabricated cytometry platform for characterization and sorting of individual leukocytes. Lab Chip 5, 30–37 10.1039/b405557h 15616737

[B29] Romagnani C. Juelke K. Falco M. Morandi B. D’Agostino A. Costa R. (2007). CD56brightCD16- killer Ig-like receptor- NK cells display longer telomeres and acquire features of CD56dim NK cells upon activation. J. Immunol. 178, 4947–4955 17404276 10.4049/jimmunol.178.8.4947

[B30] Tokimitsu Y. Kishi H. Kondo S. Honda R. Tajiri K. Motoki K. (2007). Single lymphocyte analysis with a microwell array chip. Cytometry A 71, 1003–1010 17972305 10.1002/cyto.a.20478

[B31] Trinchieri G. (1989). Biology of natural killer cells. Adv. Immunol. 47, 187–376 10.1016/S0065-2776(08)60664-1 2683611 PMC7131425

[B32] Valiante N. M. Uhrberg M. Shilling H. G. Lienert-Weidenbach K. Arnett K. L. D’Andrea A. (1997). Functionally and structurally distinct NK cell receptor repertoires in the peripheral blood of two human donors. Immunity 7, 739–751 10.1016/S1074-7613(00)80393-3 9430220

[B33] Vanherberghen B. Manneberg O. Christakou A. Frisk T. Ohlin M. Hertz H. M. (2010). Ultrasound-controlled cell aggregation in a multi-well chip. Lab Chip 10, 2727–2732 10.1039/c004707d 20820481

[B34] Vanherberghen B. Olofsson P. Guldevall K. Sternberg-Simon M. Forslund E. Khorshidi M. A. Pacouret S. Mehr R. Önfelt B. (2012). “NK cell surveillance studied over time at the single cell level by a novel microchip-based assay reveals surprising heterogenetiy in the cytotoxic response,” in Poster and Talk Presented at the 13th Meeting of the Society for Natural Immunity, Asilomar, CA

[B35] Varadarajan N. Julg B. Yamanaka Y. J. Chen H. Ogunniyi A. O. McAndrew E. (2011). A high-throughput single-cell analysis of human CD8(+) T cell functions reveals discordance for cytokine secretion and cytolysis. J. Clin. Invest. 121, 4322–4331 10.1172/JCI58653 21965332 PMC3204845

[B36] Wiklund M. (2012). Acoustofluidics 12: biocompatibility and cell viability in microfluidic acoustic resonators. Lab Chip 12, 2018–2028 10.1039/c2lc40201g 22562376

[B37] Wiklund M. Onfelt B. (2012). Ultrasonic manipulation of single cells. Methods Mol. Biol. 853, 177–196 10.1007/978-1-61779-567-1_14 22323148

[B38] Yamamura S. Kishi H. Tokimitsu Y. Kondo S. Honda R. Rao S. R. (2005). Single-cell microarray for analyzing cellular response. Anal. Chem. 77, 8050–8056 10.1021/ac0515632 16351155

[B39] Yamanaka Y. J. Berger C. T. Sips M. Cheney P. C. Alter G. Love J. C. (2012). Single-cell analysis of the dynamics and functional outcomes of interactions between human natural killer cells and target cells. Integr. Biol. (Camb.) 4, 1175–1184 10.1039/c2ib20167d 22945136

[B40] Yawata M. Yawata N. Draghi M. Partheniou F. Little A. M. Parham P. (2008). MHC class I-specific inhibitory receptors and their ligands structure diverse human NK-cell repertoires toward a balance of missing self-response. Blood 112, 2369–2380 10.1182/blood-2008-03-143727 18583565 PMC2532809

